# Educational video while “waiting-to-be-seen” in a cardiology outpatient clinic promotes opt-in self-consent for biobanking of remnant clinical biospecimens: A randomized-controlled trial

**DOI:** 10.1017/cts.2023.518

**Published:** 2023-04-11

**Authors:** Javier E. López, Andrew Kyle, Armon J. Hosseini, Machelle Wilson, Stephanie Soares

**Affiliations:** 1 Division of Cardiovascular Medicine at UC Davis, CA, USA; 2 Division of Internal Medicine at UC Davis, CA, USA; 3 Clinical Translational Science Center, University of California Davis Medical Center, Sacramento, CA, USA

**Keywords:** Biorepository, self-consent, video consent, passive consent, recruitment, opt-in consent, remnant biospecimen, biobanking, media consent

## Abstract

**Objectives:**

Consenting donors for remnant clinical biospecimen donation is critical for scaling research biorepositories. Opt-in, low-cost, self-consenting for donations that solely relied on clinical staff and printed materials was recently shown to yield ∼30% consent rate. We hypothesized that adding an educational video to this process would improve consent rates.

**Methods:**

Randomized patients (by clinic day) in a Cardiology clinic received either printed materials (control) or the same materials plus an educational video on donations (intervention) while waiting to be seen. Engaged patients were surveyed at the clinic checkout for an “opt-in” or “opt-out” response. The decision was documented digitally in the electronic medical record. The primary outcome of this study was the consent rate.

**Results:**

Thirty-five clinic days were randomized to intervention (18) or control (17). Three hundred and fifty-five patients were engaged, 217 in the intervention and 158 in the control. No significant demographic differences were noted between treatment groups. Following an intention-to-treat analysis, the rate of opt-in for remnant biospecimen donation was 53% for the intervention and 41% for the control group (*p*-value = 0.03). This represents a 62% increase in the odds of consenting (OR = 1.62, 95% CI = 1.05–2.5).

**Conclusion:**

This is the first randomized trial showing that an educational video is superior to printed materials alone when patients are self-consenting for remnant biospecimen donation. This result adds to the evidence that efficient and effective consenting processes can be integrated into clinical workflows to advance universal consenting in medical research.

## Introduction

The use of human tissues available in biobanks is critical in advancing biomedical research, science, and medical care, including the recent development of precision medicine [1,2]. Developing and sustaining annotated human sample biobanks are important components of evolving translational research infrastructure [3]. To effectively support this infrastructure, biobanks must obtain appropriate consent from donors, acquire good quality specimens and research data, maintain specimens in proper storage conditions that retain the tissue integrity, and make these specimens available for future research on-demand [4,5]. With the growth of personalized medicine, new biomarkers, and novel therapeutics, it is critical that researchers have access to sustainable biobanks with strong collections of patient biospecimens and annotated clinical data [3,6].

To develop a sustainable infrastructure for research biobanking at our institution, our Clinical Translational Science Center (CTSC) team, in collaboration with our Cardiology outpatient clinic, recently developed a fully integrated broad consent process that required very little institutional effort [7]. BURRITO or the Biospecimen Use for Research-Related Investigations and Translational Objectives is a new *IRB-approved,* self-consenting workflow for remnant sample biobanking that models local clinical workflows for ease and sustainability. It uses printed materials to inform patients at the time of a routine clinic visit and records consent for biobanking digitally with eSign directly into the Epic® electronic medical record (EMR). The low burden of this approach to the clinic makes it an attractive option for scaling up broad access to samples with opt-in consent. However, BURRITO was shown to efficiently document an opt-in consent rate ∼30% without needing research staff in the clinic [7]. This <50% rate consent, however, has tempered the enthusiasm for wider application of the process.

In this study, we hypothesized that consent rates using the established BURRITO workflow [7] would be improved with the addition of an educational video. While using audio-video (AV) media such as videos for consenting is not new [8–11], this is the first time that video-assisted education and self-consent during a routine clinic visit have been tested for broad consent of remnant biobanking. Using a randomized clinical trial design, we compared the established BURRITO workflow (control) to an improved version (intervention) that added an educational video. The primary outcome of this study was the difference in consent rates between groups. An increase of >20% odds of consenting with intervention would be considered a positive trial result. The new workflow with a video remains a low-burden approach for scaling up a front-end, opt-in consenting strategy in an ambulatory clinic.

## Methods

To test our hypothesis, we randomized subjects to follow the original BURRITO workflow with minor modifications as described below or a new BURRITO workflow (same workflow as in control with printed materials + an educational video) for consenting to a remnant biobank donation. This study is a single-center prospective, randomized controlled trial conducted during 35 prespecified days from January 30 to March 30, 2019, at the University of California Davis (UCD) outpatient general Cardiology clinic. The UC Davis Institutional Review Board (IRB) approved all aspects of the study including a waiver of consent for patients participating in the trial.

### Study Population

Patients were included if they were >18 years of age and attending a routine appointment during one of the designated study days. Only English-speaking patients were approached for this study in a pragmatic way: staff had the discretion to defer showing the materials to patients if they required translators for their visit, the physician was ready to see the patient at the time of rooming, or if patients indicated they were limited on time. Inform consent written materials other than video were available upon request in English, Spanish, and Russian, the three most common languages spoken in our clinic. Patients were included in the analysis if they endorsed reviewing the provided materials at the time of checkout.

### Intervention

There were two randomized assignments: (1) control that used mostly the original BURRITO workflow (see modifications below), and (2) intervention that added a video-enhanced workflow. Cluster randomization occurred at the level of the clinic dates. Clinical staff were blinded to the assignment of the day until they were instructed at the beginning of clinic. Patients were blinded to the ongoing trial, and the offering to consent was treated as part of their routine visit. Research staff were available remotely for synchronous and asynchronous support during the trial.

The original BURRITO workflow [7] was used as the control arm. Briefly, this process electronically documents informed consent using an EMR functionality and existing clinical staff and workflows (Supp 1-diagram). In this workflow, patients first register for a clinical appointment; while waiting in the clinic room for the physician, they review-printed documentation in the form of a trifold brochure that introduces the concepts of clinical biospecimen research and a four-sided laminate brochure that includes the consent and HIPAA information (Supp 2-laminated figure); finally, patients opt-in with self-consent at the clinic check out.

Minor modifications to the previously published workflow [7] were made with input from the clinical staff for this study as follows: (1) clinic registration staff distributed the trifold paper brochure at the registration counter; (2) laminated brochure was permanently located in obvious but unobtrusive locations within each of the clinic rooms; (3) the rooming staff-directed patients to the laminated brochure and/or video for them to review while waiting to be seen; (4) at discharge, check out staff confirmed with patients that they had viewed the materials prior to soliciting an eSignature to document their consent in the EMR.

The intervention arm followed the same procedure as controls with the addition of playing an educational video. Prior to exiting the room and after directing attention to the laminated brochure, staff would activate the animated video stored on the exam room desktop computer. This was played automatically, and the patient was not asked if they would like to watch the video. After the video, the patient had the opportunity to review the physical handouts while waiting for the physician. Neither the physician nor clinic staff directly reviewed the consent documents with subjects. Questions about the research project were referred to a remote research coordinator for asynchronous responses. At visit conclusion, the check-out staff inquired about the patient’s interest in signing the consent document in the EMR (eSignature) if they endorsed reviewing the materials.

### Video Preparation

As a collaboration between the UCD CTSC and the UC Biomedical Research Acceleration, Integration, & Development program, the Biorepositories Core Resource unit designed a 2 min and 37 s. animated video [12] for the intervention. The purpose of the video was to better inform patients on the value of biobanking remnant clinical biospecimens for future research and the collection of EMR clinical data for the study. The video was commercially produced by a third-party contractor post-IRB approval of the narrative text. The approved text contained all IRB-mandated components of informed consent. Elements throughout the video were branded to UCD Health and included Sacramento landmarks for regional customization.

### Data Collection, Analysis, and Outcome Measures

Three response options on consent were offered: “yes” or opt-in, “no” or opt-out, and “Defer Decision” which left both the “Yes” and “No” fields empty. If a “yes” or “no” to consent was elicited, this decision was immediately and electronically captured into a discrete field in the EMR. If deferred, this was documented separately, outside of the EMR; the yes/no field was then blank which would lead to asking again about their preference during the next visit. The outcome was extracted from EMR stored data via an automated weekly report for monitoring of staff and study workflow compliance.

We tested for demographic differences (sex, age, race, ethnicity, insurance payer, marital status, visit diagnosis, language and subspecialty clinic attended) between the treatment groups to validate the randomization. Assuming an alpha of 0.05 and a two-sided test, we calculated that we would need 226 subjects selecting “yes” or “no” for consent (∼113 per treatment group) to detect *a* > 20% difference in consent rates between a control group and the intervention group with a 90% power. Assuming a 30% deferral on answering the consent question, a 5% unbalanced between groups, and a nominal rate per day for engaged subjects of ∼10 subject per clinic day (all factors are based on prior experience), we aimed to recruit ∼350 subjects during 35 clinic days where the days were randomly assigned to deliver either the control or the intervention when engaging patients for consent.

The primary outcome of the study was the patient consent rates. To calculate the consent rate in an intention-to-treat analysis, we divided the number of patients who decided “yes” by the total number of patients engaged in that arm of the study because the randomization occurred at the level of the clinic day. As a secondary outcome, we calculated the “as-treated” consent rate by dividing the number of patients who decided “yes” by the sum of patients who decided “yes” and “no” while excluding those that deferred consenting.

### Statistical Analysis

We used Fisher’s exact test to test for differences between categorical variables and the Satterthwaite *t* test to test for differences in age between the outcome and treatment groups. A *p*-value of less than 0.05 was considered statistically significant. All analyses were performed using SAS® software version 9.4 (SAS Institute Inc., Cary, NC).

## Results

**Fig. 1.**
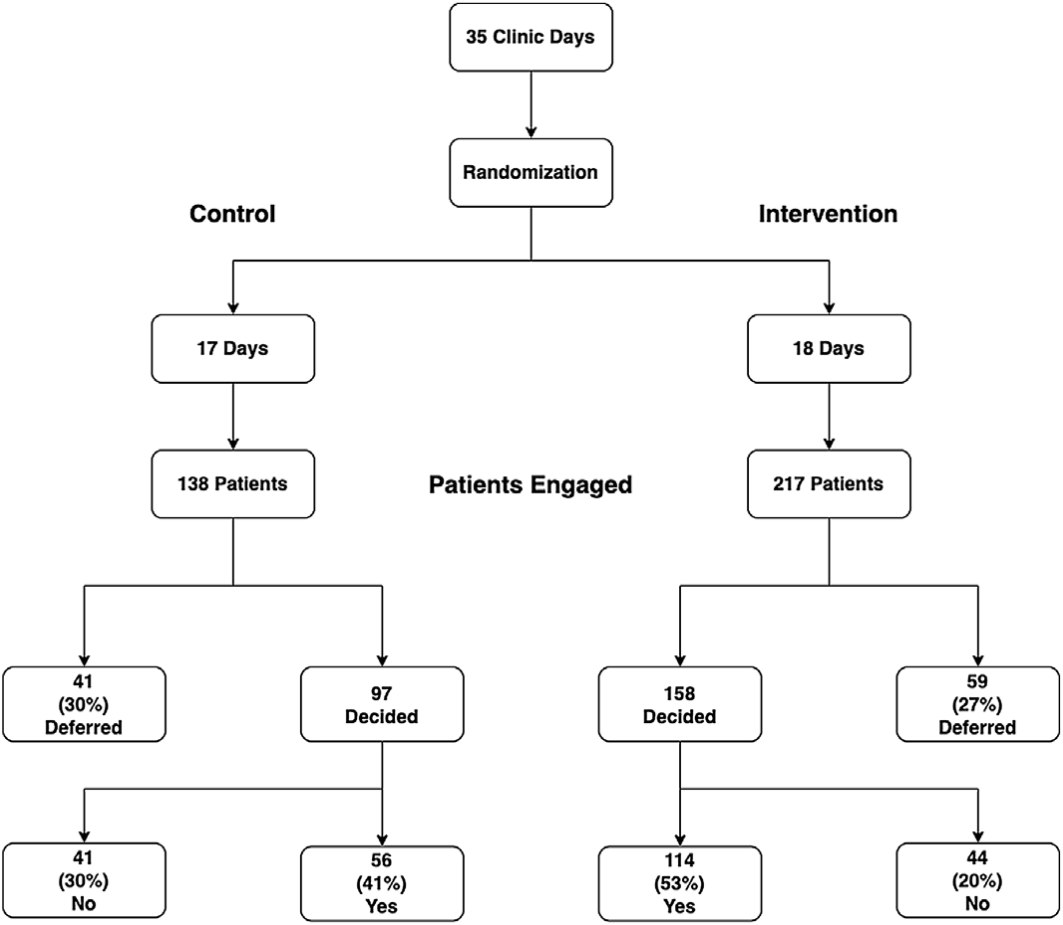
Flow diagram illustrates the progression of subjects through study and outcomes for both control and intervention groups. Randomization began with clinic days and transitioned into patients represented in each arm. This is followed by a display of the results by deferment and decision of the patients in both control and intervention; corresponding consent rates are shown based on an intention-to-treat analysis.

Thirty-five clinic days were randomized for this study. Seventeen days were designated to the control arm, and 18 days were for the intervention. Responses were elicited in a total of 355 patients (Fig. 1). In the days designated to the control arm, 138 (39%) patients were engaged, and of these, 41 (30%) patients deferred responding. In the intervention group, 217 (61%) patients were engaged, and of these, 59 (27%) deferred answering.

For the intention-to-treat analysis, 56 (56/138, 41%) control patients opted in (yes) and 41 (41/138, 30%) patients opted out (no). In the intervention arm, 114 (114/217, 53%) opted in (yes) and 44 (44/217, 20%) opted out (no). This difference in opting in rates represents a 62% increase in the odds of consenting (OR = 1.62, 95% CI = 1.05 to 2.5, *p*-value = 0.03) with the use of the educational video. For the as-treated analysis, 72% (114/158) of the intervention patients opted in and 28% (44/158) opted out. Meanwhile, only 58% (56/97) of the control patients opted in and 42% (41/97) opted out (OR = 1.96, 95% CI = 1.15 to 3.34, *p*-value = 0.014).

There were no significant differences between the treatment groups in any of the measured demographics (Table 1). Eight out of 255 subjects (3%) were labeled in the EMR as non-English speakers but were deemed appropriate to participate by the clinic staff based on their level of English comprehension on the day of the study.

**Table 1.** Demographic differences between treatment and control groups in randomized subjects who decided during the study. Data are reported as *percent (n) for categorical variables*, or as *mean (standard deviation)* for numeric variables as indicated

Variables	Intervention arm (*N* = 158)	Control arm (*N* = 97)	*p*-Value
Mean age (years)	65.4 (14.1)	66.4 (12.2)	0.56
Sex (female)	48.1% (76)	46.4% (45)	0.80
Payor			
Private Medicare MediCal	43.0% (68) 56.3% (89) 0.63% (1)	41.2% (40) 56.7% (55) 2.1% (2)	0.69
Marital status			
Married	57.0% (90)	52.6% (51)	0.87
Single	15.8% (25)	17.5% (17)	
Divorced/widowed	18.4% (29)	21.7% (21)	
Other/unknown	8.9% (14)	8.3% (8)	
Race			
White Black Asian Other	71.5% (113) 5.7% (9) 5.7% (9) 17.1% (27)	73.2% (71) 10.3% (10) 1.0% (1) 15.5% (15)	0.16
Ethnicity Hispanic Not Hispanic unknown	7.6% (12) 88.6% (140) 3.8 (6)	5.2% (5) 90.7% (88) 4.1% (4)	0.81
Clinic type			
Advanced therapies Arrhythmia Faculty General	5.1% (8) 9.5% (15) 46.8% (74) 38.6% (61)	11.3% (11) 7.2% (7) 52.6% (51) 28.9% (28)	0.14

## Discussion

The main finding of this randomized control trial is that adding an educational video to an established opt-in, self-consent process in the outpatient clinic significantly increased the rate of consenting to 53% from 40.5% (*p*-value = 0.03) in the control group without video (primary outcome) with an odds ratio of 1.62 in favor of the intervention. Since randomization occurred at the level of the clinic day, an intention-to-treat analysis was used to include all patients engaged in this calculation. If the analysis is conducted assessing the consent rates among patients who were engaged and decided yes/no, an “as-treated” analysis, the consent rates between the intervention and control groups would be 72 and 58%, respectively, and an odds ratio of 1.98 favoring the intervention. Together, both analyses in this study support the superiority of utilizing a video for opt-in self-consenting.

Two additional unexpected results were noted: 1) the control group had a higher than previously reported [7] 30% consenting rate at 41%, and 2) nearly twice as many people engaged in the process when the video was used. Together, these results demonstrate that the BURRITO plus video workflow offers a path forward for low-effort, widespread implementation of an opt-in consenting process for the collection of remnant biospecimens and annotated clinical data for research.

Consenting research participants is a necessary step and an inevitable challenge for recruiting subjects into clinical studies; this challenge must be met by all prospective studies to succeed. Failed recruitments can lead to inconclusive results and/or biased representation of distinctive populations. Meeting recruitment targets is a predicament that affects many research settings. While individual reports differ, estimates suggest that the proportion of clinical studies meeting their original targets is likely to be <50% [11,13]. One review of 114 trials [13] found that fewer than one-third of trials recruited to 100% of their original target, and 45% recruited below 80% of target. In this cohort of trials, the most reported strategy to improve recruitment was providing additional study information to clinical staff and patients. Our study did just that by adding an educational video [12] to an already existing process for self-consenting in our Cardiology outpatient clinic [7]. Our new consent process was built to obtain broad consent of remnant biospecimens. In this report, we now show it to be highly effective in this setting. While this type of research is certainly different than clinical interventional trials, it is reasonable to hypothesize that our consent approach could also be tested in other settings in the future.

Our results contrast with two Cochrane reviews that have recently covered or referenced the topic of AV media use when consenting for clinical studies [10,11]. While they concluded that little or no difference to consenting rates occurs from utilizing AV media, we must caution that most of the available evidence was of low to very low quality and rarely tested in randomized control trials where consenting rates were the primary outcome [8–11,14]. Many other studies in the literature are retrospective analyses, and most of them call for additional prospective trials like the one reported here [10,11]. Often, primary outcomes in reported studies are limited to patient understanding or satisfaction rather than consent rates. Consent rates are critical to determine efficacy and efficiency for operational planning and sustainability of the research infrastructure. Study interventions and controls also vary significantly, for example, video versus face-to-face consent with research staff, or consents by interactive research tablets versus pamphlets which could be challenging for the translation of results to practice. Rarely, randomized control trials are used to compare head-to-head superiority between two methods of consenting as done in the current study. Furthermore, we utilized existing clinical staff and clinic infrastructure to provide and collect the consent (i.e., video was played in existing clinic computers by clinic staff and consent documented in the EMR at discharge by the clinic staff) which makes our intervention easily and efficiently translatable to any clinic without the need of research hardware or staff.

Two unexpected results were noted in our study. Both unexpected results contributed to the main goal of our study. First, the control group had a 11% higher consent rate than previously reported with the original BURRITO workflow [7]. The current trial was conducted in the same clinic where the first BURRITO workflow was built, and there, the nominal consenting rate was 30%. In preparation for this trial, the clinical staff in the clinic were encouraged to improve the original BURRITO workflow for efficiency following principles of quality improvement [15]. Minor adjustments (noted in the methods section) were added to the protocol, and the clinic staff received a second training hour to review the modifications. These quality adjustments are believed to be responsible for the enhanced consent rate of the control arm; despite this higher-than-expected rate in the control arm, the intervention still demonstrated superiority when compared to control.

Second, nearly twice as many patients engaged in the process when the video was used. In fact, 79 extra patients decided on consent in the 18 days of intervention in contrast to the 17 days of control. The intervention group was allotted one extra clinic day during randomization which could only account for ∼10 extra patients based on the average of patient engaged per day. Hence, this extra 30% of patients engaged is most likely attributed to the video in the intervention. The mechanism for this difference is unclear. Two possibilities come to mind: 1) the staff were more engaged and attentive when presenting the video, and/or 2) the patients were more engaged and attentive when reviewing materials after the video. While we have no data to support either of these possibilities, we noted that the fraction of patients deferring in both groups was similar (30% vs. 27%), and their demographics and clinic diagnosis were also not significantly different. These observations suggest that differences in consenting are not likely due to staff bias in selecting the patients to present the video, but rather something related to the experience of viewing the video that drove a larger number of patients to respond to the inquiry. However, this hypothesis will need to be tested in additional studies. This is a remarkable complement to the increased opt-in consenting rate (primary outcome); together these findings make the use of BURRITO plus video a very attractive option for scaling up and sustaining enterprise-wide consenting for biobanking of remnant samples with minimal additional effort.

Regardless of the mechanism, this additional 30% engagement in the consent process occurred without significant additional day-to-day burden on resources or cost to the enterprise. The major investment of time and resources necessary to implement this process is at the front end (20–30 hours total effort). This requires a project manager with an understanding of the clinic infrastructure who is available to answer questions and provide teaching sessions to the clinic staff. An EMR clinical analyst is also needed to design the changes within the check-out process, which requires approximately 2 weeks of work (40–60 hrs.). Once deployed, project analysts can track quality performance and rate of participation as an ongoing project. While this process was designed and implemented in 2019, we do not anticipate any significant changes in a post-COVID world for physical clinic interactions. However, the post-COVID transition to a higher percentage of virtual and phone appointments that cannot provide the setting to show the video during the wait time in the clinic may be a barrier to implement this process. Because this transition has occurred in many locations, capturing these remote patients will require developing new methods for self-consenting. Nevertheless, we still believe that adding a video to other educational materials will be helpful even if the workflow occurs remotely, but this was not tested directly in this study.

### Limitations

One limitation to our study is that we only included English-speaking subjects. Limiting the video to English comprehension was done to accelerate testing of the program in production, but we only sample this subgroup of patients in the clinic. Hence, our results should not be extrapolated to non-English speakers without further testing on how to bridge language and/or understanding gaps. Translating the video and written materials to the patient’s native language, we propose, will be critical for the success of biobanking programs that aim to include all people in their collections.

A second limitation of our study is that we did not assess understanding or acceptability of our process and/or biospecimen research in our patient population. Numerous other factors beyond language such as cultural differences, comfort with the medical system, and/or trust to protect privacy can affect the consent rates but were not tested in this study. Because a long history of skepticism and mistrust exists among some patients regarding biospecimen research, we provided an e-mail address and phone numbers for participants to contact our coordinator and ask questions about the process and/or the research itself. Only five individuals out of 355 (1.4%) called to ask further questions. We received no complaints about the study or the materials; in the contrary and anecdotally, the staff reported repeated praises for the process. However, we did not test for understanding or acceptability directly and suggest that this should be evaluated in the future as a quality assurance for this new process.

## Conclusion

The broader purpose of our intervention was to improve the consent process for the recruitment of patients to biobanking research. Our data support that implementing BURRITO plus video into the self-consent process is efficacious and efficient for widespread translation and scaling without significant burden to the health care system. Implementation will require addressing language barriers and the world of remote care for widespread access. This new process was also well received by staff and patients without obvious operational disruptions.

## Supplementary material

For supplementary material accompanying this paper visit https://doi.org/10.1017/cts.2023.518. click here to view supplementary material
