# Supplementary material for: Educational video while “waiting-to-be-seen” in a cardiology outpatient clinic promotes opt-in self-consent for biobanking of remnant clinical biospecimens: A randomized-controlled trial
Source: J Clin Transl Sci. 2023 Apr 11;7(1):e103. doi: 10.1017/cts.2023.518 (PMC10225258; doi:10.1017/cts.2023.518)
Supplement: Supplementary file 1 [file S2059866123005186sup001.docx]

Supplemental Index

Table of Contents

1. Diagram of Original BURRITO workflow………………………………………………………………1
2. Informational Research Brochure……………………………………………………………………….2
3. Diagram of adapted BURRITO workflow with intervention…………………………………3

Supplemental Figure 1. Diagram of Original BURRITO Workflow.


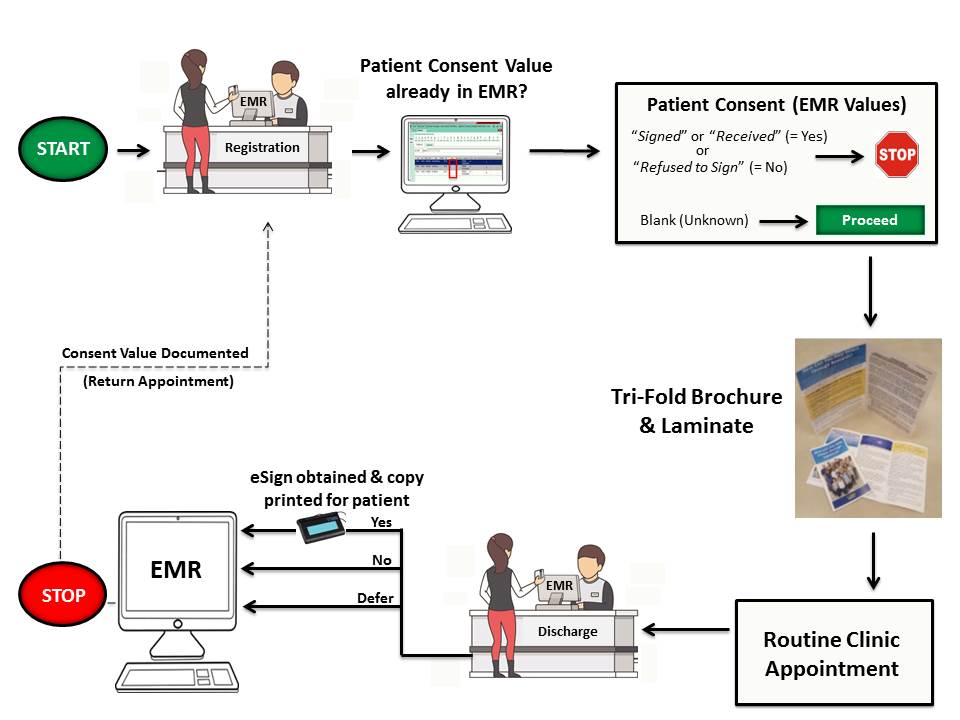


Supplemental Figure 2. Informational Brochure on Biorepository Research

Supplemental Figure 3. Diagram of adapted BURRITO workflow with intervention
